# An Integrated Approach to Using Sheep Wool as a Fibrous Material for Enhancing Strength and Transport Properties of Concrete Composites

**DOI:** 10.3390/ma15051638

**Published:** 2022-02-22

**Authors:** Rayed Alyousef, Hossein Mohammadhosseini, Ahmed Abdel Khalek Ebid, Hisham Alabduljabbar

**Affiliations:** 1 Department of Civil Engineering, College of Engineering, Prince Sattam bin Abdulaziz University, Al-Kharj 11942, Saudi Arabia; h.alabduljabbar@psau.edu.sa; 2 Institute for Smart Infrastructure and Innovative Construction (ISIIC), School of Civil Engineering, Universiti Teknologi Malaysia (UTM), Skudai 81310, Malaysia; mhossein@utm.my; 3 Faculty of Engineering and Technology, Future University in Egypt, New Cairo 11845, Egypt; Ahmed.AbdelKhaleq@fue.edu.eg

**Keywords:** concrete composites, sheep wool fibers, transport properties, strength, microstructure

## Abstract

An important goal to achieve sustainable development is to use raw materials that are easily recyclable and renewable, locally available, and eco-friendly. Sheep wool, composed of 60% animal protein fibers, 10% fat, 15% moisture, 10% sheep sweat, and 5% contaminants on average, is an easily recyclable, easily renewable, and environmentally friendly source of raw material. In this study, slump testing, compressive and flexural strengths, ultrasonic pulse velocity, sorptivity, and chloride penetration tests were investigated to assess the influence of wool fibers on the strength and transport properties of concrete composites. Ordinary Portland cement was used to make five concrete mixes incorporating conventional wool fibers (WFs) ranging from 0.5 to 2.5% and a length of 70 mm. The wool fibers were modified (MWFs) via a pre-treatment technique, resulting in five different concrete compositions with the same fiber content. The addition of WF and MWF to fresh concrete mixes resulted in a decrease in slump values. The compressive strength of concrete was reduced when wool fibers were added to the mix. The MWF mixes, however, achieved compressive strength values of more than 30 MPa after a 90-day curing period. Furthermore, by including both WF and MWF, the flexural strength was higher than that of plain concrete. In addition, adding fibers with volume fractions of up to 2% reduced the concrete composite’s sorptivity rate and chloride penetration depths for both WF and MWF content mixes. Consequently, biomass waste like sheep wool could be recycled and returned to the field following the circular economy and waste valorization principles.

## 1. Introduction

The circular economy is becoming a driving force behind the development of new technologies and the modernization of old production cycles. As is well known, the circular economy is built on utilizing prospective waste materials in new productive chains and procedures, such as energy generation or the development of novel materials. The main goals are to avoid exploiting non-renewable raw resources and avoid the cost and effect of waste disposal. This technique can effectively address some of the most pressing social and environmental issues we face today; for example, climate change, the rise in produced wastes resulting from rapid population growth, and resource shortages [1,2,3,4]. Nowadays, improperly managed farming systems pose several environmental hazards [4].

Sheep wool is another notable example of a product tied to farming. Sheep are notable for food security because they are proficient and can adapt to a wide range of climates, from the frigidity of Iceland to the wastelands of Africa, Asia, and Australia [5]. Several studies on using natural fibers such as sheep wool in the construction industry have focused on its use as a thermal and acoustic insulating material [1,6]. Like “mineral wool” and calcium silicate, sheep wool is equivalent to other insulating materials. Sheep wool fibers are recognized as suitable thermal and sound absorption materials, according to the results of various experimental measurements [7,8,9]. Sheep wool fibers also have a good hygroscopicity nature, allowing them to absorb moisture, avoid condensation, and control humidity in insulation materials [10]. Wool is naturally flame-retardant due to its high water and nitrogen content. Sheep wool fibers are likewise good acoustic materials, but material thicknesses greater than 170 mm provide no further acoustic benefits, as stated by Zach et al. [11].

Fiber-reinforced concrete (FRC) is a conventional concrete mix that comprises cement, coarse and fine aggregates, and a random dispersion of discontinuous short fibers. The fibers enhance the concrete mixture’s ductility, energy absorption, and tensile and flexural strengths. Many types of fibers, either polymeric or metallic, are commonly used in concrete mixtures for their benefits. Glass, steel, synthetic, and natural fibers, and fibers from pre-and post-consumer wastes, recycled carbon fibers, and recycled tire textures are the most prevalent fibers used in FRC [12,13]. Fibrous concrete with pozzolanic materials has also been created and investigated in conjunction with conventional concrete. There is no doubt that new supplementary cementitious materials (SCMs) have recently grown in popularity, providing exceptional physical, mechanical, and durability features [14]. These SCMs are utilized all around the world, according to Mousavi et al. [15], for their technical, economic, and ecological benefits. Fibers in cement matrices promote material toughness by enhancing fracture propagation resistance. In this regard, incorporating fibers, depending on their aspect ratios, might help delay the initiation of cracks [16]. The majority of these fibers are artificial, have an environmental impact due to large carbon emissions, and are not very cost-effective. Environmentally friendly building materials using natural fibers from various sources are receiving interest in the research community. Another advantage is that they are light and easy to process. They are also environmentally friendly and reusable [17]. The mechanical properties of FRC, such as compressive, bending, and tensile strengths, modulus of elasticity, ultimate strain fracture toughness, impact and seismic resistance, ductility, and durability, are all governed by fiber–matrix interactions. According to research, FRC with exceptional mechanical qualities cannot be created unless the fiber–matrix interfacial bond is at least equal to the matrix’s tensile strength. However, a particularly strong fiber–matrix link does not ensure the development of ductile FRC, highlighting the relevance of matrix and fiber characteristics. The fiber’s tensile strength, matrix microstructure, and length/alignment concerning the applied stress/content of the fibers all have a role in the fiber’s capacity to resist breaking [18,19].

Natural fibers include lignocellulosic, mineral, and protein fibers; using protein fibers such as sheep wool and animal hair is limited in concrete production [20]. Furthermore, using waste products like building materials will save money over the long-term. Khan and Ali [21] investigated a concrete pavement using human hair as a fibrous material and found that using 0.8% of these fibers in the concrete resulted in a 3% cost saving per lane per kilometer of road and a 12% rise in compressive strength. Wool and hair obtained from animals were found to have similar physical qualities, structures, and uses to most textile fibers. Sheep wools are required to be trimmed off for the health care of sheep, and cannot be discarded openly owing to environmental considerations; 2.3–3.6 kg raw wool is produced every year on average [22]. However, recycling of sheep wool fibers and use as a construction material after minor treatment has been investigated, as these fibers have a high elastic modulus of up to 4 GPa [4]. In addition, sheep wool has increased strength due to chemical interaction between the protein components [23].

Despite the advantages mentioned above, the long-term durability of cement-based composite materials containing waste sheep wool fibers limits large-scale production [24,25]. However, there is limited evidence on the long-term durability of concrete composites with wool fibers in any previous research. The durability issue is related to the pH of the cement matrix’s effect on the sheep wool fibers. Jóźwiak-Niedźwiedzka and Fantilli [4] found that the alkalinity ratio of the cement matrix directly affected the long-term performance of wool fibers in mortar. In a cement matrix with high alkalinity and humidity, sheep wool fibers lose their strength and negatively affect the bridging action of fibers across the cracks. This therefore results in a reduction in the strength of the matrix. Two major strategies have been used to reduce the degradation of wool fibers in cement-based composites: pre-treatment of wool fibers and modification of the cement matrix to lower the alkalinity [3]. Consequently, to reduce the alkalinity of the concrete matrix and improve durability performance, the utilization of supplementary cementing materials, such as pozzolanic materials, and the fibers’ surface modification, have been recommended [26,27]. 

Although this research includes an investigation of waste sheep wool fibers available, the conducted experiments and analyses are based on one single type of fiber. The work has been focused on the performance of concrete containing conventional and modified wool fibers, but it is believed that technical issues have to be understood and corrected before utilizing any type of waste fibers in concrete. Furthermore, effective pre-treatment can increase the physical properties of wool fibers, hence improving the mechanical properties and durability of concrete composites. In addition to conventional concrete, the use of discarded natural fibers in fiber-reinforced concrete is increasingly being studied in specific applications. To date, and to the authors’ best knowledge, there are limited studies on the strength and transport characteristics of concrete containing waste sheep wool fibers. Consequently, due to the local availability of waste sheep wool, these fibers are used to produce concrete composites to examine their influences on the mechanical and transport properties.

## 2. Experimental Program

### 2.1. Materials and Sample Preparation

**Figure 1:**
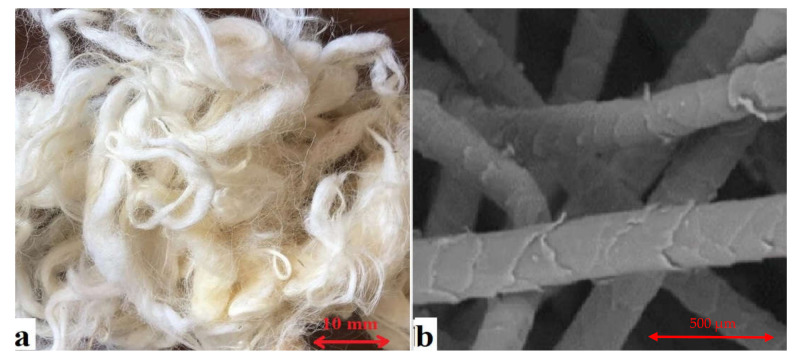
(**a**) Natural sheep wool fibers and (**b**) SEM image of sheep wool fibers.

In this study, type I ordinary Portland cement (OPC) was used in accordance with the specifications of ASTM C150-07. For fine aggregates, clean and dry natural river sand was used, which was passed through a 4.75 mm sieve, had a specific weight of 2.6, fineness modulus of 2.3, and water absorption of 0.70%. Crushed granite with a specific weight of 2.7, 0.5% water absorption, and a maximum size of 20 mm was employed as coarse aggregate. Furthermore, the waste sheep wool fibers (WFs) used in this investigation were obtained from local sources in Saudi Arabia. In this work, WF with a length of 70 mm was employed, as illustrated in Figure 1. Table 1 lists some of the most commonly utilized WF properties. The influence of WFs as fibrous materials on the performance of concrete with a constant water-to-cement ratio (W/C) of 0.50 was investigated using nominal concrete mixtures.

The ACI-211-19 standard was used for proportioning concrete mixtures. The concrete mixes were divided into two groups of regular wool fiber (WF) concrete mixes and modified wool fiber (MWF) concrete mixes with fiber volume fractions of 0.5%, 1%, 1.5%, 2%, and 2.5%, respectively, based on the total volume of concrete mix referred to as WF1–WF5 and MWF1–MWF5. Additionally, a batch of concrete without any fiber was made as a control mix (C). This range of fiber dosages was chosen based on the available literature and a series of preliminary experimental studies conducted prior to the main work. However, fiber additions greater than 2.5% have a detrimental effect on the performance of concrete. Following the existing literature [2,3,7,8] and also a set of trial experiments, the pre-treatment and modification of wool fibers were performed by immersing the conventional sheep wool in a plastic tank filled with salty water for 24 h at room temperature to increase the fiber’s surface friction and provide a robust bonding strength between fibers and the cement matrix. The pre-treatment solution was made by diluting freshwater with 35% salt. 

The process of mixing ingredients into fiber-reinforced concrete is somewhat different from that of normal concrete. Cement binders were added to the dry mix of fine and coarse aggregates in the first step. Then, water was added and mixed for approximately two minutes. Finally, the required volume of fiber was added to the mixture, and the mixing process was continued for another two minutes to ensure that the fibers were evenly dispersed in the mix. Table 2 summarizes the proportions of concrete mixtures. After casting, the specimens were cured for 24 h at a room temperature of 20 ± 5 °C. The samples were then de-molded and stored in a water tank until the testing day. 

### 2.2. Testing Methods

**Figure 2:**
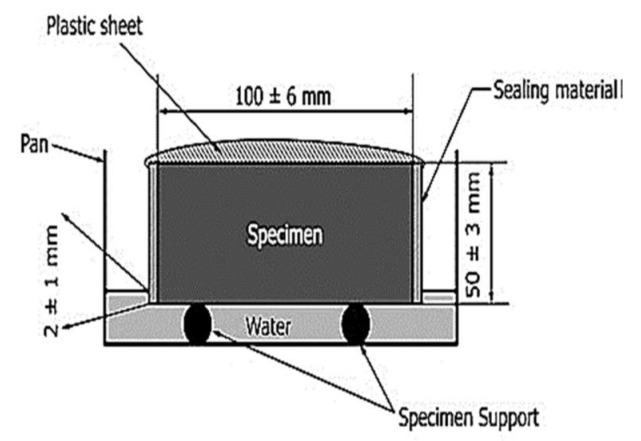
Sorptivity test setup following ASTM C1585-13 specifications.

The workability test was performed on plain and reinforced concrete mixtures following ASTM C143-03 to determine the slump values. Compressive strength tests were conducted on cylindrical specimens of size 150 mm in diameter and 300 mm in height in accordance with ASTM C39-19. The ultrasonic pulse velocity (UPV) test was performed before the compressive strength test. Prism beam samples with a size of 150 mm × 150 mm × 700 mm were prepared according to ASTM C78-18 to conduct the flexural strength test. Cylindrical samples of size 100 mm × 200 mm were used for the chloride penetration and sorptivity tests. The sorptivity test of concrete specimens reinforced with wool fibers was determined according to ASTM C1585-13 requirements, as illustrated in Figure 2. Concrete disks of 100 mm in diameter and 50 mm in height were cut from 100 mm × 200 mm cylinders. The specimens were then dried for 72 h at a constant temperature of 50 ± 2 °C. Following that, the concrete specimens were stored for another fifteen days in a sealable container. Next, the original weight of samples was noted to the nearest 0.01 g. Three samples were taken from each batch of concrete, and the average of these three results was used to measure the water absorption rate.

**Figure 3:**
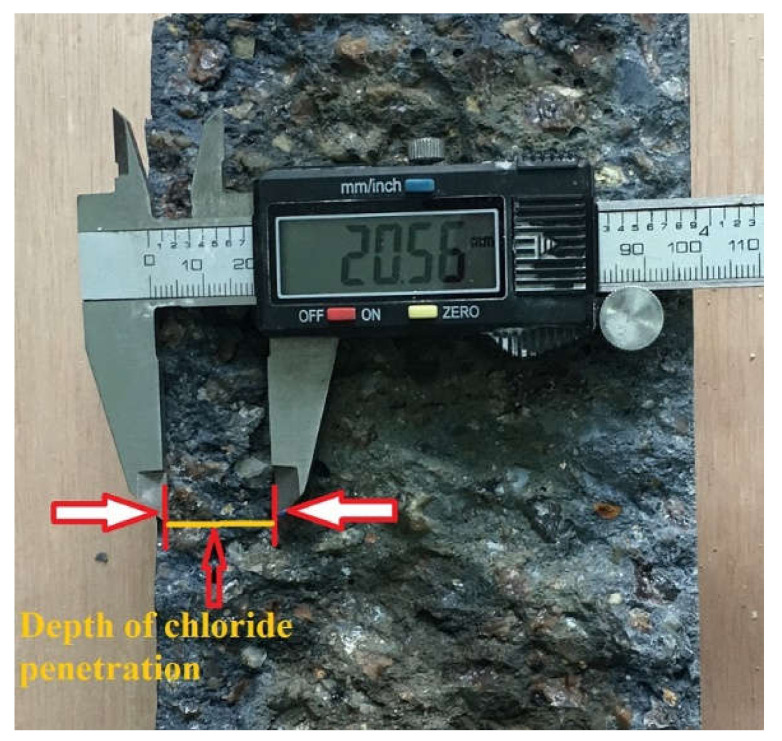
Depth of chloride penetration after spraying with AgNO_3_ solution.

A solution with 5% sodium chloride (NaCl) was prepared to imitate harsh conditions in which concrete members can deteriorate significantly when exposed to seawater, and the concrete samples were immersed in it to test the chloride penetration depth. After the exposure periods of 7, 28, and 90 days had been completed, the cylindrical specimens were cut diagonally down the length of the concrete cylinders into two parts. The concrete samples’ split surface was then sprayed with 0.1 N silver nitrate (AgNO_3_) solution to determine the chloride penetration depth. After spraying the AgNO_3_ solution onto the concrete specimens, the outer portion was brighter due to the silvery deposit of silver chloride (AgCl), which indicated the depth of chloride penetration, whereas the innermost part was darker owing to the occurrence and consequence of silver hydroxide (AgOH), as shown in Figure 3.

## 3. Results and Discussion

### 3.1. Workability

**Figure 4:**
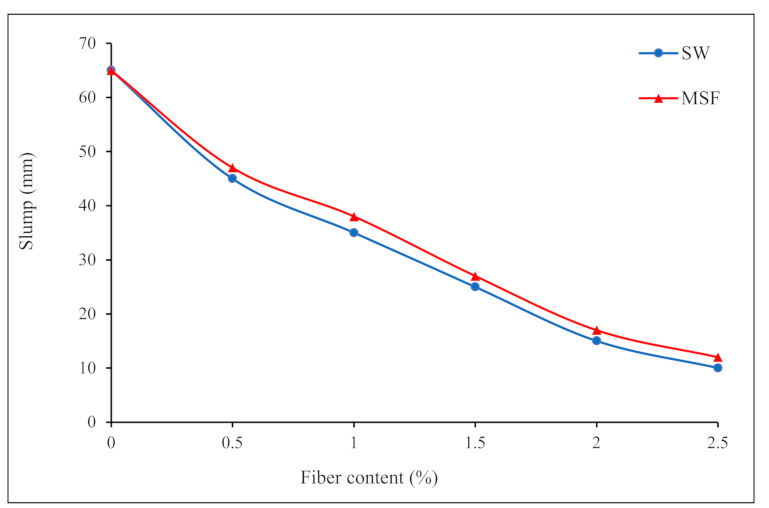
Effects of conventional wool fibers (WFs) and modified wool fibers (MWFs) on the workability of fresh concrete mixes.

Despite the advantages of fibers in concrete mixtures, they can make fresh concrete difficult to work with and place in molds, compromising the hardened state’s performance. This is because the relative mobility of coarse aggregate particles is affected by the dimensional compatibility of fibers. Furthermore, adding fibers results in absorbing the free water in the concrete matric due to the large surface area of the fibers, which lowers the quantity of water available to lubricate cement particles and, consequently, reduces the flowability of fresh concrete [28]. The slump test was carried out to assess the workability of fresh concrete mixtures containing sheep wool fibers. The addition of sheep wool fibers considerably affected the flowability of concrete mixes, as seen in Figure 4. The conventional concrete mix without any fibers had a slump of 65 mm. The slump values dramatically decreased when sheep wool fibers were added to the concrete mix; for instance, at a fiber dosage of 2.5%, the slump values were about 10 mm, indicating that adding wool fiber to the concrete made the mixture harsher and less workable.

Furthermore, for concrete with higher fiber content, the fibers can tangle and form balls during the mixing process, called the “balling effect”, making it difficult to achieve the appropriate workability of the mixture, resulting in lower slump values. This is because the inclusion of WF can create a network structure in the matrix, preventing segregation and allowing the concrete to flow freely. As a result, increasing the fiber dosage will make it more difficult to keep the mixes flowing. However, a higher initial slump contributes to the complete fluidity of the mixtures at low shear rates, implying that the concrete mix will lose less workability in this situation [29,30]. The results revealed that the slump values of mixtures comprising modified wool fibers (MWFs) were slightly greater than those of conventional wool fiber (WF) mixes. This could be linked to the MWF treatment, which reduced water absorption from fresh concrete and increased workability.

### 3.2. Compressive Strength

**Figure 5:**
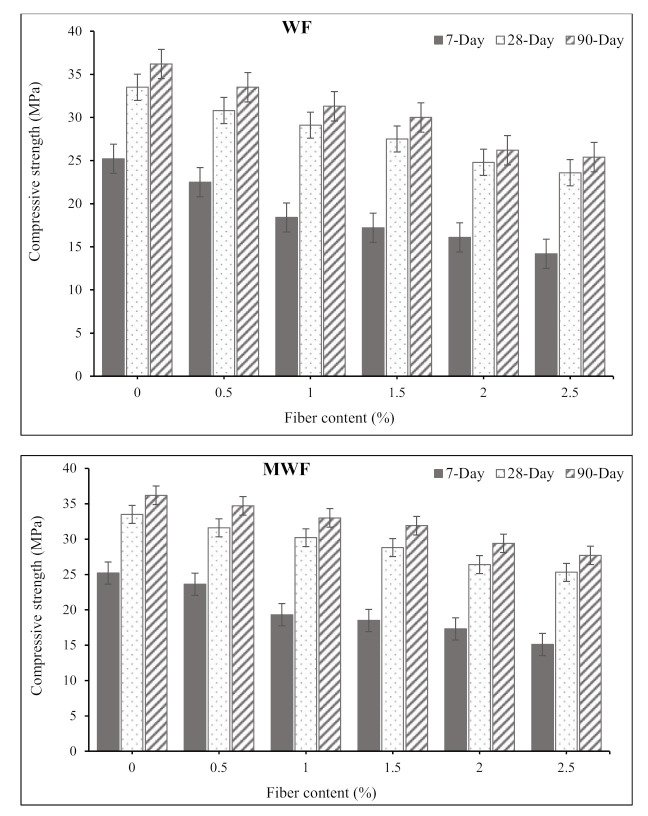
Variation in the compressive strength of concrete mixtures by adding wool fibers.

**Figure 6:**
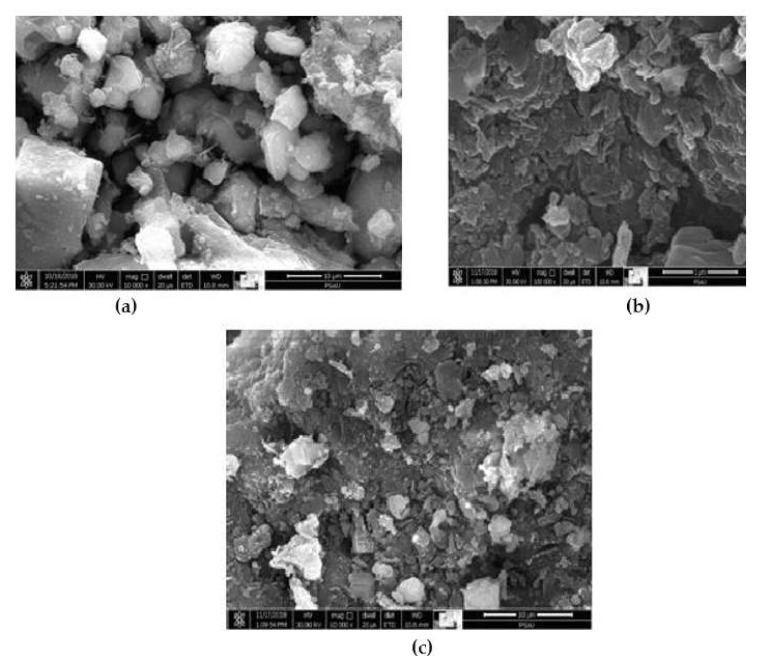
SEM images of (**a**) control mix, (**b**) mix containing 1% WF, and (**c**) mix containing 2% WF.

Figure 5 shows the compressive strength experimental findings for concrete mixes reinforced with WF and MWF. The results reveal that increasing the amount of wool fiber in concrete mixes reduces the compressive strength of the mixture. It can be noted that modified wool fiber mixes have higher strength values than conventional wool fiber mixtures. The addition of WF and MWF to concrete resulted in a reduction in the concrete’s compressive strength values. The compressive strength of WF specimens decreased by about 7.5%, 13.4%, 17%, 27.6%, and 29.8%, respectively, after 90 days of curing, when sheep wool fibers were added to the concrete mixtures at dosages of 0.5%, 1%, 1.5%, 2%, and 2.5%. Likewise, compressive strength values decreased by roughly 4.1%, 8.8%, 11.9%, 18.7%, and 23.5% in specimens reinforced with the same dosages of MWF fibers. It can be seen that the rate of strength reduction in the MWF mixes is marginally lower than in the WF mixes. The reduction in strength could be due to the concrete compositions’ low workability, resulting in the uneven distribution of fibers in the matrix. The amount of fiber in the concrete matrix could determine the air void content, affecting the concrete strength. In addition, the rise in porosity can be associated with the growth of micro-cracks in the specimen and, therefore, poor fiber–matrix bonding and lower strength [30,31,32]. Figure 6 demonstrates that fiber additions resulted in greater pore size and lower strength, indicating these internal physical changes.

Because the density of wool fibers is comparatively lower than that of plain concrete, adding fibers beyond 1% made the concrete a soft matrix and resulted in lower density and compressive strength of the concrete, which is attributed to the variances amongst the density of reinforcement and the cement matrix [16]. The results of this study are in agreement with those findings reported by Gradinaru et al. [33] on the combined effects of fly ash and waste sheep wool fibers on the compressive strength of concrete. They reported that the addition of sheep wool fibers into concrete mixtures did not improve the compressive strength, where the recorded strength values were lowered by 15–30% when sheep wool fibers were added compared to a plain concrete mix. In addition, Valenza et al. [34] studied the mechanical properties of a cement mortar reinforced with sheep wool fibers, and they reported that mixtures reinforced with wool fibers had lesser strength values than the plain mixture, independent of the amount and length of fibers.

### 3.3. Flexural Strength

**Figure 7:**
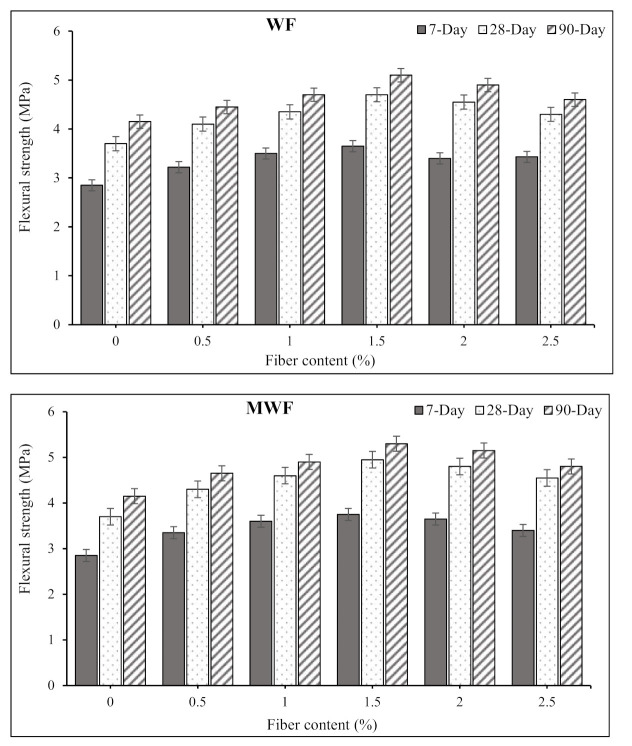
Variation in the flexural strength of concrete mixtures by adding wool fibers.

Natural fibers can be used to reinforce concrete and mortar, increasing their ductility and providing a more sustainable alternative to standard industrial fibers. Furthermore, such fibers can bridge the surfaces of fissures in the post-cracking stages, reducing the building industry’s environmental impact [13]. Figure 7 shows the results of flexural strength tests of concrete mixtures reinforced with varied dosages of WF and MWF at various ages. The addition of wool fibers raised the flexural strength of concrete mixtures significantly at all doses, as can be seen. At 90 days of curing, the flexural strength values for the concrete mix reinforced with 0.5%, 1%, 1.5%, 2%, and 2.5% WF were 4.45, 4.7, 5.1, 4.9, and 4.6 MPa, respectively, which are all greater than the 4.15 MPa obtained for the control plain concrete mix. It can be seen that the concrete mixture reinforced with 1.5% WF had the highest flexural strength. Further increases in fiber content, however, led to reduced flexural strength values. This phenomenon could be due to non-uniform fiber distribution. The inter-particle friction between fibers and aggregates also affects the orientation and distribution of the fibers and therefore the strength properties of the concrete composite. The increase in porosity may be linked to micro-cracks, poor fiber–matrix bonding, and lower strength [31,35,36].

The concrete mixtures reinforced with MWF showed a similar tendency in flexural strength improvement, although the achieved values were higher than those reported for mixes containing WF. For example, at 90 days, the flexural strength values for the same fiber doses were 4.65, 4.9, 5.3, 5.15, and 4.8 MPa, respectively, which are all greater than the 4.15 MPa observed for the control plain concrete mix. The fibers improved concrete components’ cracking response and deflection capacity before failure, resulting in increased ductility and energy absorption overall. Additionally, increased fiber content caused cracks to be bridged up to a reliable load limit. 

**Figure 8:**
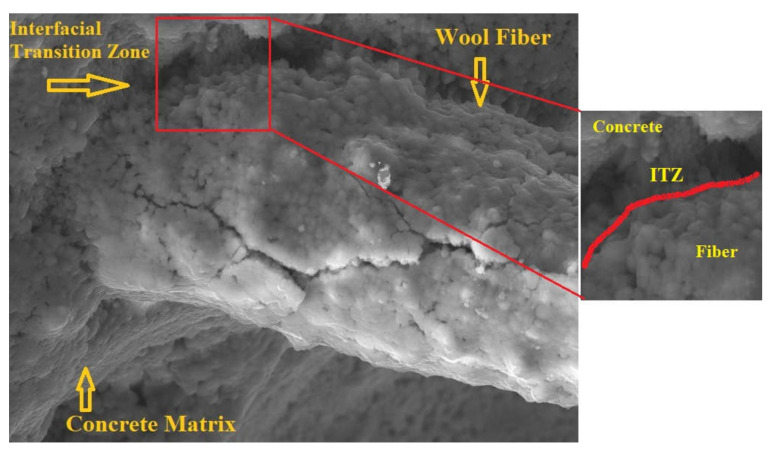
The interfacial transition zone between wool fiber and concrete matrix.

Several approaches have been developed to improve the fiber–matrix interfacial connection. These strategies can be divided into three categories: macroscopic changes to mechanical properties and fibers, microscopic changes to fiber surface characteristics, and microscopic matrix augmentation with microfibers or transition zone densification. All of these strategies have the potential to improve the fiber–matrix bond significantly. For metallic fibers, however, the transition zone densification approach is appropriate. The majority of the enhancing approaches have so far been developed at the microscale level of the FRC constituent materials [17,21]. Figure 8 reveals the interfacial transition zone (ITZ) between concrete matrix and wool fibers. The SEM image shows a robust and uniform bond amongst the concrete matrix and fibers. The fiber coverage of cement hydration products is also highly evident, indicating that the fiber–matrix adhesion is good. A denser matrix in the ITZ can result in a stronger bond, resulting in increased flexural strength and ductility.

As a result of the high fiber content in the reinforced specimens compared to the plain samples, the flexural strength improved. Greater fiber dosages caused better fiber spreading over the matrix, putting it in the path of crack development. The lower strength in the higher fiber dosages could be related to inadequate fiber dispersion within the concrete matrix, which causes fiber agglomeration in the matrix. Fantilli et al. [22] also made similar observations. They looked at how sheep wool fibers affected the mechanical qualities of mortar. Their research demonstrated that adding wool to cementitious mortar formulations increased flexural strength and ductility. Maia Pederneiras et al. [37] also found that using wool fibers in cement mortars increased flexural strength, whereas the fibers with a length of 30 mm showed a more significant increase in flexural strength than those of shorter fibers. 

### 3.4. Ultrasonic Pulse Velocity

**Figure 9:**
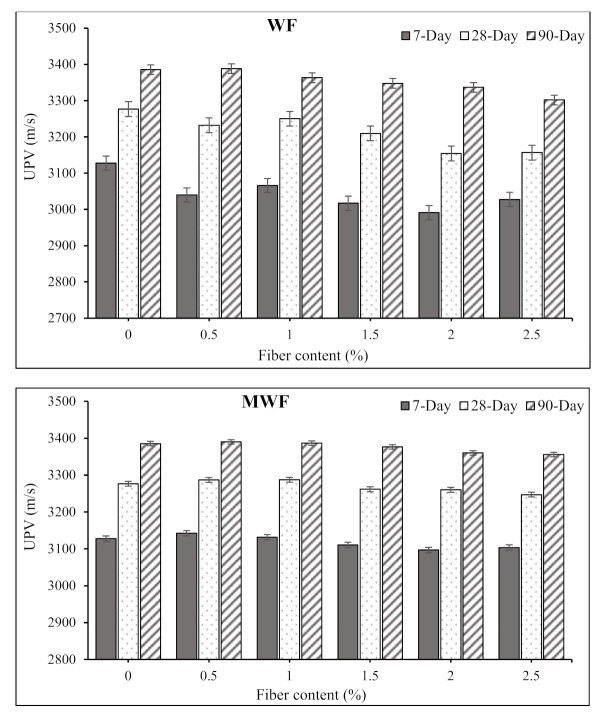
Effects of wool fibers on the UPV values of concrete mixtures.

Figure 9 shows the observed ultrasonic pulse velocity (UPV) values of concrete mixtures at various curing ages. According to the findings, the presence of wool fibers has no significant effect on the UPV values, especially at an early age. The addition of wool fibers raised the UPV values for extended curing times. At 28 and 90 days, UPV values of 3300 m/s to 3400 m/s were recorded for plain concrete mixes and concrete mixes reinforced with WF and MWF. Concrete mixtures are categorized as good quality according to the recorded UPV values higher than 3300 m/s [38]. The addition of up to 1% MWF fibers results in greater UPV at longer curing periods. For instance, at 90 days, the observed UPV for specimens comprising 0.5% and 1% MWF are 3390 and 3387 m/s, respectively, higher than the 3385 m/s for the plain concrete mix. The UPV of concrete with fiber content greater than 1%, however, was found to be reduced. The presence of pores in the matrix, which reduce the consistency with high volume percentages of fibers, is well known to cause a decrease in the UPV of concrete [8,38].

**Figure 10:**
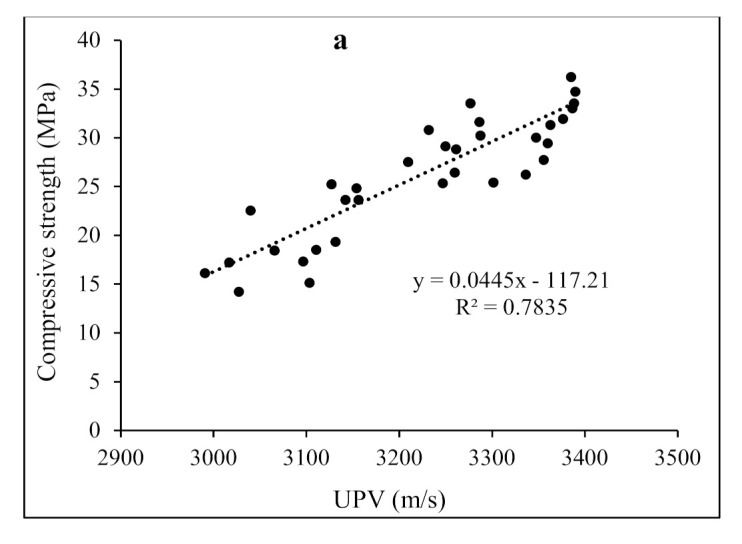

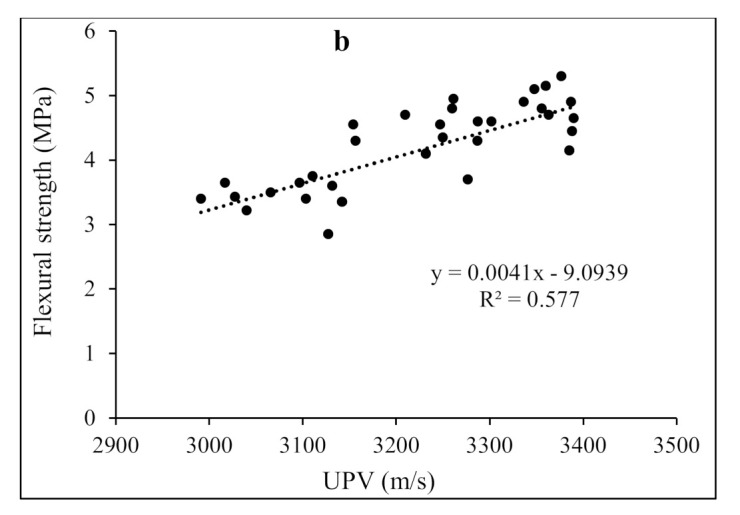
Correlations between (**a**) compressive strength, (**b**) flexural strength and UPV values.

Additionally, some factors such as type of aggregates, curing period, W/C ratio, size of specimens, and the testing procedures can influence the UPV values of concrete specimens in the same way as compressive and flexural strength might. Other factors, such as the types and shape, the aspect ratio, and the dosage of wool fibers, could affect the strength of concrete in addition to the considerations mentioned above. The velocity of compression waves is affected by the strength and other mechanical parameters of concrete in the UPV technique. The density and elasticity of the material also affect the velocity of these waves. As a result, the UPV is useful for determining concrete quality and uniformity, estimating static and dynamic modulus of elasticity, and measuring time-dependent concrete behavior [38]. In addition, examining the number of concrete samples is vital since a wider diversity of investigational data can deliver the most accuracy for statistical validation of various issues. As a result of the collected experimental results, empirical relationships correlate the strength properties and UPV values of concrete mixtures reinforced with WF and MWF. A regression analysis was used to determine the impact of WF and MWF on the characteristics of concrete. A linear regression analysis was utilized to reveal the correlations between the compressive and flexural strengths and the obtained UPV values, as shown in Figure 10. Figure 10a depicts a link between compressive strength and UPV values, while Figure 10b illustrates a relationship between flexural strength and UPV, yielding Equations (1) and (2) with coefficients of determination (R^2^) of 0.783 and 0.577 for all samples.

*f_c_* = 0.0445*V* − 117.21   R^2^ = 0.7835 (1)

*f_f_* = 0.0041*V* − 9.0939   R^2^ = 0.577 (2)

where *f_c_* and *f_f_* are the compressive and flexural strengths (MPa), respectively, and *V* is the UPV (m/s).

**Figure 11:**
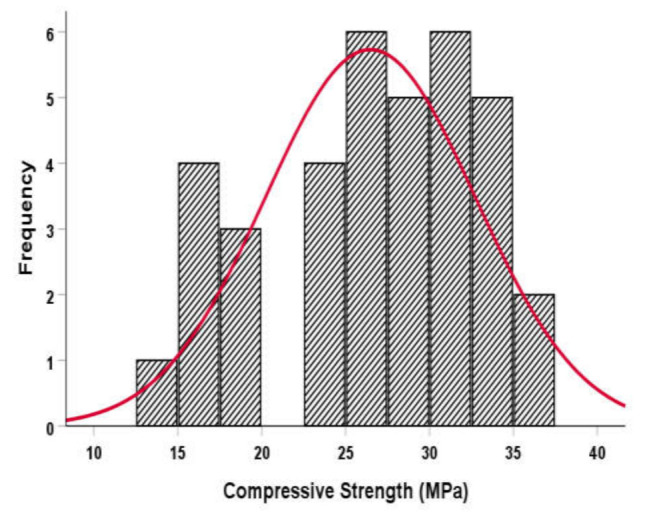

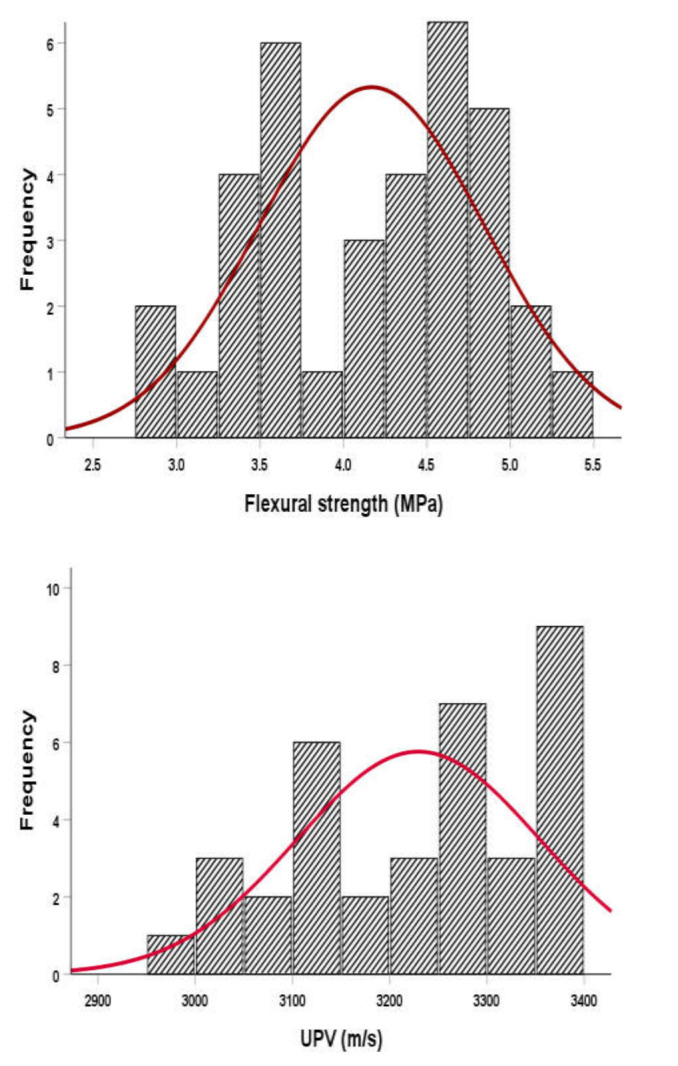
The histogram of the strength and UPV values of concrete mixtures reinforced with wool fibers.

The compressive and flexural strengths and UPV values of concrete mixtures comprising WF and MWF were examined, as well as their probability distribution and analytical investigation. The mechanical properties of polypropylene and steel-fiber-reinforced concrete are the subjects of most statistical data analysis in the literature. However, no statistical research has been performed on the mechanical characteristics and UPV of concrete reinforced using sheep wool fibers. As a result, statistical analysis is required to understand better the properties of concrete based on the experimental results. The impact of fiber content on the statistical considerations and distributions of flexural and compressive strengths and UPV is substantial and deserves to be discussed. Therefore, the statistical software SPSS was used to statistically calculate the mechanical characteristics and UPV. The compressive strength, flexural strength, and UPV values for sheep wool fiber-reinforced concrete mixes are revealed in Figure 11. The attained *p*-values were greater than 0.05 for all tests according to the statistical analysis and findings. At the 0.05 level of significance, this result verifies the null hypothesis. Consequently, the mechanical characteristics and UPV of concrete mixtures are distributed normally. The current study’s findings are comparable to those reported by Mastali et al. [39].

### 3.5. Sorptivity

**Figure 12:**
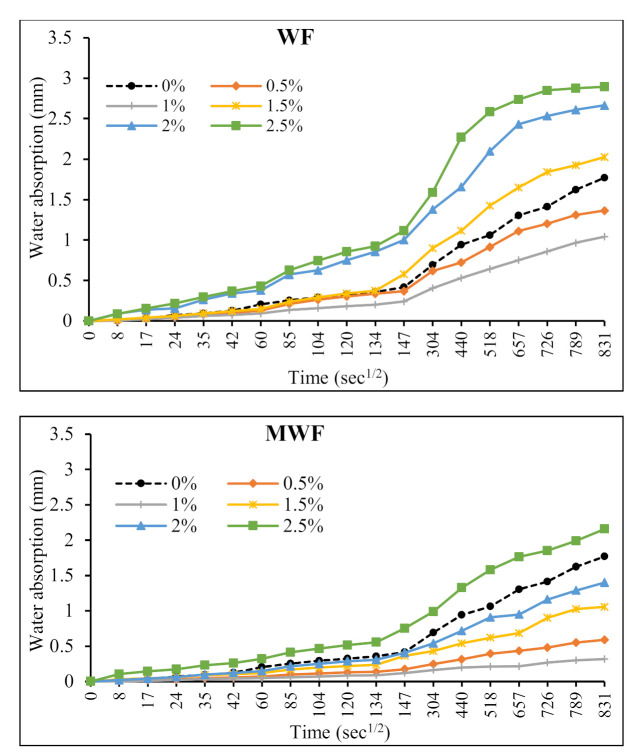
Effect of wool fibers on the rate of water absorption of concrete mixtures.

Exploitation circumstances and external factors determine the durability of wool-fiber-reinforced concrete composites. The quality and developments that appear at the fiber/matrix interface determine the bonding amongst fibers and the cement matrix, which is a critical factor. Their high proclivity limits natural fibers’ utility in cement-based composites for degrading in an alkaline environment. When fibers are utilized as reinforcement in concrete subjected to harsh environments, they frequently lose strength [40]. The results of a sorptivity test for concrete mixtures reinforced with WF and MWF are shown in Figure 12. The addition of WF up to 1% and MWF up to 2% lowered the water absorption of the mixtures when compared to plain concrete without any wool fibers.

When fiber dosages of more than 1% and 2% for WF and MWF, respectively, were added, water absorption increased slightly. The results showed that the water absorption rate in reinforced specimens was lower than in their conventional equivalent mix, up to a specific fiber dose. Lower water absorption in reinforced specimens indicates lower environmental harm, confirming the matrices’ lower permeability. With the addition of fibers beyond 2%, due to the uneven distribution of fibers in the concrete matrix, the volume of pores increased, consequently reducing the concrete density and providing more space in the matrix to absorb water and a higher water absorption rate [41,42]. The findings show that raising the fiber volume fraction changed the distribution and size of air voids in the matrices. In this regard, Garca et al. [43] stated that the volume of air voids in a concrete matrix is primarily determined by the percentage of masses of fibers in the matrix. In contrast, De Schutter et al. [44] confirmed that a higher porosity volume in the concrete matrix causes increased sample degradation due to a higher water absorption rate. Shawnim et al. [42] found that an uneven spreading of large holes might increase permeability, leading to greater water absorption.

### 3.6. Chloride Diffusion

**Figure 13:**
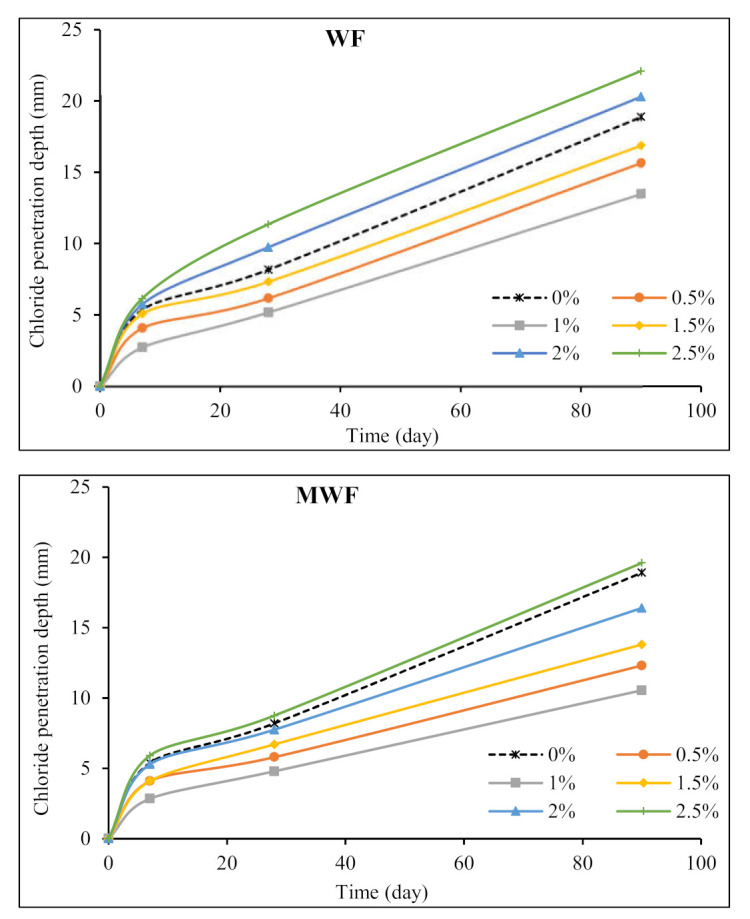
Effects of wool fibers on the chloride penetration depth of concrete mixtures.

Although sheep wool fibers can withstand almost any solvent, alkalis can cause them to disintegrate even in diluted solutions. Temperature increases accelerate the dissolution reaction, which damages fibers when the pH is above 11. Wool fibers can be damaged by both acids and alkalis [3,45,46]. Wet wool’s strength is mainly determined by the covalent cross-links formed by disulfide bonds. As a result, pre-treatment and altering wool fibers may be viable options for increasing their chemical resistance. The chloride penetration depth test was performed on concrete specimens containing WF and MWF by immersion in a 5% chloride solution, with the outcomes shown in Figure 13.

Wool fiber reinforcing of concrete mixtures up to 1.5% generated a grid and dense structure in the concrete matrix that substantially influenced reducing chloride diffusion into specimens and the formation of microcracks. According to the test results, the penetration depth in those mixtures containing WF and MWF at doses of 0.5%, 1%, and 1.5% was significantly decreased. The lowest depth of chloride penetration was 10.5 mm for concrete reinforced with 1% MWF at 90 days, roughly 44% lower than the noted value of 18.9 mm for the plain concrete mix. However, increasing the fiber content raised the permeability of the matrix, which increased the penetration depth. When relating the penetration depths for concrete mixes, it can be seen that pre-treatment and modification of wool fibers resulted in a better performance in preventing the entry of chemicals into the concrete matrix, resulting in the formation of a grid structure and a decrease in the chloride penetration rate when compared to mixes comprising normal wool fibers. The characterization of the interface, which plays a vital role in the durability of concrete components, is critical for service life modeling and prediction. Generally, fiber-reinforced concrete with high dosages of fibers has porous interfaces with lower strength. Increased binding strength and loss of flexibility of fiber bundles are directly linked to the durability of natural fibers such as wool fibers [47,48]. Pre-treating fibers to increase their properties and utilizing admixtures to provide a strong link between fibers and matrix are two alternate options for improving the performance of concrete composites exposed to harsh conditions.

## 4. Conclusions

Sheep wool fibers are one of the most sustainable natural resources owing to ease of recyclability, low carbon impact, biodegradability, and energy-efficient properties. Therefore, the current study determined the feasibility of reinforcing the concrete mixtures with conventional and modified waste sheep wool fibers, and proposed novel concrete composites. The mechanical and transport properties of concrete specimens comprising various dosages of normal and modified wool fibers were investigated. Based on the experimental and analytical results, the following conclusions can be drawn:

- The addition of wool fibers and increased fiber dosages remarkably reduced the workability of concrete mixtures, at which the slump values dropped to about zero at a fiber content of 2.5%.
- When sheep wool fibers were added, the compressive strength was slightly reduced. Nevertheless, pre-treatment wool fibers resulted in higher strength values than normal wool fibers. The obtained compressive strength values ranged between 28 and 35 MPa for the mixes containing 0.5% to 2.5% MWF at the age of 90 days, which signifies the sufficient strength that can be utilized for various applications.
- Despite the reduction in compressive strength of concrete mixtures, considerable increases in flexural strength of all concrete mixes were recorded. All reinforced mixes obtained greater flexural strength values than plain concrete mixes. The maximum flexural strength of 5.3 MPa was obtained for the concrete mix containing 1.5% MWF at the age of 90 days, which is about 28% higher than that of 4.15 MPa recorded for the plain control mix.
- The addition of wool fibers did not significantly affect the UPV values. However, the obtained UPV values of higher than 3300 m/s classified the concrete as good quality concrete.
- Pre-treatment of sheep wool fibers with saltwater resulted in significantly improved fiber characteristics and better adherence to cement paste. In addition, a lesser percentage of modified sheep wool fibers improves mechanical qualities, whereas the same improvement in strength can be achieved with a higher rate of untreated wool.
- The addition of wool fibers up to a specific dosage reduced the water absorption rate of concrete mixtures and resulted in a lower rate of chloride penetration in reinforced specimens. This was owing to the fiber’s bridging action and providing a rigid network, which prevents the entrance of liquids into the concrete matrix. In addition, wool fibers can absorb up to 28% of their weight in water without feeling wet, which significantly interacts with the concrete matrix in reducing the water absorption rate.
- The overall performance of concrete mixtures may be improved by a particular treatment of sheep wool fibers. Consequently, concrete composites comprising modified waste sheep wool fibers are regarded as one of the most promising sustainable building materials for environmentally friendly construction with comparatively lower cost.

## Figures and Tables

**Table 1:** Properties of sheep wool fibers.

Fiber	Diameter (µm)	Length (mm)	Aspect Ratio	Tensile Strength (MPa)	Water Absorption Capacity (%)	Ultimate Tensile Strain (%)
Sheep wool fibers	95–130	70	550–650	390	28	50.2

**Table 2:** The proportions of the concrete constituents.

Mix ID	V_f_ (%)	WF (g/m^3^)	OPC (kg/m^3^)	Fine Agg. (kg/m^3^)	Coarse Agg. (kg/m^3^)	Slump (mm)
C	0	0	8	16	24	65
WF1	0.5	40	8	16	24	45
WF2	1.0	80	8	16	24	35
WF3	1.5	120	8	16	24	25
WF4	2.0	160	8	16	24	15
WF5	2.5	200	8	16	24	10
MWF1	0.5	40	8	16	24	47
MWF2	1.0	80	8	16	24	38
MWF3	1.5	120	8	16	24	27
MWF4	2.0	160	8	16	24	17
MWF5	2.5	200	8	16	24	12

## Data Availability

The data presented in this study are available on request from the corresponding author. The data are not publicly available due to size of the research.
